# Pyroptosis-Related Signature Predicts Prognosis and Immunotherapy Efficacy in Muscle-Invasive Bladder Cancer

**DOI:** 10.3389/fimmu.2022.782982

**Published:** 2022-04-11

**Authors:** Qi Zhang, Yezhen Tan, Jianye Zhang, Yue Shi, Jie Qi, Daojia Zou, Weimin Ci

**Affiliations:** ^1^ Key Laboratory of Genomic and Precision Medicine, Beijing Institute of Genomics, China National Center for Bioinformation, Chinese Academy of Sciences, Beijing, China; ^2^ University of Chinese Academy of Sciences, Beijing, China; ^3^ Department of Urology, Peking University First Hospital, Beijing, China; ^4^ Institute of Urology, Peking University, Beijing, China; ^5^ National Urological Cancer Center, Beijing, China; ^6^ Institute for Stem Cell and Regeneration, Chinese Academy of Sciences, Beijing, China

**Keywords:** muscle invasive bladder cancer, pyroptosis, PRGScore, prognostic model, immune

## Abstract

Pyroptosis has profound impacts on tumor cell proliferation, invasion, and metastasis and is of great clinical significance for different cancers. However, the role of pyroptosis in the progression and prognosis of muscle invasive bladder cancer (MIBC) remains poorly characterized. Here, we collected multicenter MIBC data and performed integrated analysis to dissect the role of pyroptosis in MIBC and provide an optimized treatment for this disease. Based on transcriptomic data, we developed a novel prognostic model named the pyroptosis-related gene score (PRGScore), which summarizes immunological features, genomic alterations, and clinical characteristics associated with the pyroptosis phenotype. Samples with high PRGScore showed enhancement in CD8^+^ T cell effector function, antigen processing machinery and immune checkpoint and better response to immunotherapy by programmed cell death 1 (PD-1) and programmed cell death ligand 1 (PD-L1) inhibitors, which indicates that PRGScore is a valuable signature in the identification of populations sensitive to immune checkpoint inhibitors. Collectively, our study provides insights into further research targeting pyroptosis and its tumor immune microenvironment (TME) and offers an opportunity to optimize the treatment of MIBC.

## Introduction

Bladder carcinoma (BLCA) is one of the most common leading causes of cancer-related mortality worldwide, accounting for approximately 573,278 new cases and more than 212,536 related deaths each year (1). The number of individuals diagnosed with BLCA and BLCA-related deaths has shown an increase in the United States over the last five years (2, 3). Most bladder cancers are urothelial carcinomas and are classified into 2 subtypes according to muscle invasiveness: muscle invasive bladder cancer (MIBC) and non-muscle invasive bladder cancer (NMIBC) (4–6). MIBC with tumor stages T2 to T4 accounts for most patient mortality (7) and is a heterogeneous disease characterized by abundant chromosomal alterations, a high mutation rate and an increased probability of metastasis (8). Compared to NMIBC, MIBC is more aggressive and is associated with a 5-year survival rate of <50% for patients with localized disease and <10% for patients with distant metastases despite radical surgery (9).

Even though perioperative platinum-based chemotherapy improves the overall survival compared with surgery alone (10), existing treatments for MIBC are insufficient because recurrence and metastasis impede clinical management and decrease the survival of many patients. Recently, the approval of immune checkpoint inhibitors (ICIs) for platinum-refractory patients with advanced urothelial carcinoma has changed the treatment paradigm (11). However, the fraction of bladder cancer patients sensitive to ICIs is limited, and they are of specific MIBC subtypes according to previous studies (12–14). Although many biomarkers, including signatures based on gene expression (15), DNA methylation (16) and copy number variation (CNV) (17), have been shown to be prognostic in MIBC, it is still unclear to what extent they will influence the clinical practice and whether they could serve as an indication of responsiveness to immunotherapy. Therefore, there is an urgent need for the development of an effective gene signature for risk stratification and to guide clinical treatment, especially with regard to targeted therapy and immunotherapy.

Pyroptosis is an inflammatory form of programmed cell death involving caspases, granzyme proteases, and pore-forming gasdermins (18). The protein family of gasdermins consists of gasdermin A-E and pejvakin which are encoded by *GSDMA*, *GSDMB*, *GSDMC*, *GSDMD*, *DFNA5* (*GSDME*) and *PJVK* (*DFNB59*) (18, 19). Cleavage of full-length GSDMs at the linker region liberates their N-terminus from the inhibitory C-terminus, thus allowing them to oligomerize at the plasma membrane to form pores and induce pyroptotic cell death (20). The cleavage is mediated by canonical caspases and granzymes, including CASP1, CASP3, CASP4, CASP5, CASP6, CASP7, CASP8, CASP9, GZMA and GZMB (18–20). Caspase-3 is an apoptotic caspase that can be activated by either intrinsic or extrinsic apoptotic pathways, where the former involves permeabilization of the mitochondrial membrane and the assembly of apoptosomes, leading to activation of caspase-9, and the latter requires activation of death receptors and caspase-8 (21). In addition to caspases, GZMB, a serine protease, can be released by cytotoxic lymphocytes, including natural killer (NK) cells and CD8^+^ T cells, to trigger pyroptotic cell death of target cells by the cleavage of cytosolic GSDME (22). Previous studies have revealed several sophisticated cleavage mechanisms for different pore-forming proteins. Examples are the cleavage of GSDME by either GZMB or caspase-3 (22, 23), the cleavage of GSDMD by human caspase-1, caspase-4 or caspase-5 (18), and the activation of GSDMB by apoptotic caspase-3, -6 and -7 but not inflammatory caspases (24). In addition, GZMA, another cell death-inducing protease expressed in cytotoxic lymphocytes, is capable of cleaving GSDMB, which is expressed in epithelial tumors of the digestive tract, to mediate pyroptotic cell death (25).

Several studies have demonstrated the influence of pyroptosis on tumor cell proliferation, invasion, metastasis and patient prognosis in various types of cancer (26). Novel pyroptosis-related gene signatures were also proposed for predicting the prognosis of ovarian cancer (27), lung adenocarcinoma (28) and gastric cancer (29). In bladder cancer, the USP24/GSDMB/STAT3 axis is reported to promote tumor proliferation and growth, where USP24 interacts and stabilizes GSDMB, which in turn binds to STAT3, increases its phosphorylation and activates STAT3 signaling (30). However, the prognostic value of the pyroptosis-related signature in MIBC has not been elucidated. Here, we integrated expression profiles from The Cancer Genome Atlas (TCGA: https://www.cancer.gov/tcga), Gene Expression Omnibus (GEO) and other public datasets for exploration and comprehensive evaluation of pyroptosis signatures in MIBC. We found that the pyroptosis signature of MIBC exhibited a correlation with important molecular and clinical characteristics, including the expression of immunomodulators, activity of the cancer immunity cycle and infiltration level of tumor-infiltrating immune cells. From the pyroptosis signature, we derived three pyroptosis patterns associated with distinct overall survival (OS) and TME features, which suggested that pyroptosis played a nonnegligible role in shaping the TME of MIBC. To this end, we built a novel scoring system named PRGScore to quantify the pyroptosis state based on the expression profiles of pyroptosis-related genes. PRGScore is indicative of prognosis, immune infiltration, and immunotherapy response in MIBC. Our findings suggest a potential connection between pyroptosis, prognosis, TME, and the response to immunotherapy in MIBC.

## Materials and Methods

### Data Collection and Preprocessing

For TCGA-BLCA, RNA-seq data in fragments per kilobase of exon per million mapped fragments (FPKM) values and matched clinical data were downloaded from the UCSC Xena data portal. Then FPKM values were transformed into transcripts per kilobase million (TPM) values. Somatic mutation and CNV data were obtained by using the R package TCGAbiolinks (31). Somatic mutation data sorted in the form of Mutation Annotation Format (maf) were analyzed and then used to calculate TMB using the R package maftools (32). CNV calling were performed with GISTIC2 (33).

From GEO, we obtained 6 microarray datasets [GSE31684 (34), GSE48075 (35), GSE87304 (13), GSE169455 (36)] and 1 single-cell RNA sequencing (scRNA-seq) dataset of bladder cancer (GSE135337). The quality control, cell clustering and annotation of scRNA-seq data were performed as previously described (37). Briefly, patients with tumor stages T2 to T4 were included in our subsequent analysis.

To investigate the predictive efficacy of PRGScore on patient response to immunotherapy, we included processed gene expression of a metastatic urothelial cancer (mUC) cohort (EGAS00001002556) that received atezolizumab treatment *via* the R package IMvigor210CoreBiologies (http://research-pub.gene.com/IMvigor210CoreBiologies) (14). In addition, we obtained a mUC cohort (GSE176307) (38) that received immune checkpoint blockade (ICB) from GEO. We also obtained processed RNA-seq data in a transcripts per million (TPM) matrix of patients treated with anti-PD1 ICB from a large melanoma genome sequencing project (MGSP) (39).

### Curation of Pyroptosis-Related Genes

From literature we curated a total of 5 gasdermins (*GSDMA*, *GSDMB*, *GSDMC*, *GSDMD* and *GSDME*), 8 caspases (*CASP1*, *CASP3*, *CASP4*, *CASP5*, *CASP6*, *CASP7*, *CASP8* and *CASP9*), and 2 granzyme proteases (*GZMA* and *GZMB*) as the most relevant pyroptosis-related regulators. GSDMs A-E were included because structural studies suggested that they share highly conserved N-terminal and inhibitory C-terminal domains separated by a variable linker, which implies similar functions. However, *PJVK* was exclude because pejvakin adopts a different structure where the C-terminal domain is too short to inhibit the pore-forming function of the N-terminal domain, making its functional roles doubtful and elusive (18). In addition to GSDMs, we listed caspase-1, caspase-3, caspase-4, caspase-5, caspase-6, caspase-7, caspase-8, caspase-9, granzyme A and granzyme B since they were reported to mediate the cleavage of gasdermins, which is crucial to pyroptosis (18–20). Though caspase-11 is also important for pyroptosis, it was not considered due to its murine origin (40).

### Consensus Clustering

We identified distinct pyroptosis regulation patterns based on the expression of pyroptosis-related cleavage enzymes by using consensus clustering with the k-means method. The number of clusters and their stability were defined by the consensus clustering algorithm using the R package ConsensusClusterPlus with 1,000 repetitions (41).

### scRNA-Seq Data Analysis

We performed the quality control (QC) and cell clustering analysis on the integrated dataset based on t-SNE algorithm implemented in Seurat following the online pipeline (https://satijalab.org/seurat/) (42). CellChat (http://www.cellchat.org/) was used to analyze the intercellular communication networks from scRNA-seq data (43).

### Gene Set Variation Analysis (GSVA) and Single Sample Gene Set Enrichment Analysis (ssGSEA)

GSVA enrichment was performed with the R package GSVA (44). Pathways in Gene Ontology (GO) and Kyoto Encyclopedia of Genes and Genomes (KEGG) were downloaded as “c5.go.bp.v7.4.symbols” and “c2.cp.kegg.v7.4.symbols” from the MSigDB database (v7.4) (45–47). Signature gene sets for bladder cancer were collected from previous studies (8, 14). The 7 steps in the cancer immunity cycle reflecting the anticancer immune response were defined as previously described (48). Activities for each of these steps were estimated using ssGSEA based on the gene expression of individual samples. Differences in gene set scores among subgroups estimated based on *t* test by using limma in R (49). An adjusted *P* value < 0.05 was considered statistically significant.

### Evaluation of Immune Cell Infiltration in TME

CIBERSORTx was applied to quantify the proportions of immune cells in the TME (50). Briefly, mixture files containing TPM values were used to impute cell fractions based on the LM22 (22 immune cell types) signature matrix file. Batch correction was performed in B-mode guided by “LM22 Source GEP”, and quantile normalization was disabled.

### Identification of Differentially Expressed Genes (DEGs) and Functional Enrichment Analysis

DEGs between every 2 groups of the 3 pyroptosis patterns and between PRGScore-high and PRGScore-low groups were determined based on *t* tests by using the R package limma. An adjusted *P* value < 0.05 was considered statistically significant. Gene set enrichment analysis (GSEA) was performed using the R package clusterProfiler (51).

### Prediction of Treatment Response to Immune Checkpoint Inhibitor Therapy

Tumor immune dysfunction and exclusion (TIDE) with default parameters was employed to predict the clinical response to ICI therapy. Patients with high TIDE scores were predicted to be non-responders, while patients with low TIDE scores were considered to be responders.

### Univariate and Multivariable Regression

We performed univariate Cox regression on TCGA-MIBC with gene expression and overall survival. Multivariate Cox regression was used to evaluate independent risk factors in the same cohort. Genes and factors with a false discovery rate (FDR) < 0.05 were considered statistically associated with patient survival. The results of univariate and multivariate Cox regression were acquired and visualized by using the R package forestplot.

### Development of Pyroptosis-Related Gene Score

To build a quantification system based on pyroptosis-related genes, we started by extracting DEGs for each pair of groups in TCGA-MIBC classified by the three pyroptosis patterns. Then, common DEGs were taken, and univariate Cox regression analysis was performed to assess associations between these overlapping DEGs and overall survival in the TCGA-MIBC dataset. Prognostic DEGs were selected as pyroptosis-related genes for principal component analysis (PCA). By borrowing the concept of m6Ascore (52), a scoring algorithm named PRGScore was developed for the quantification of the pyroptosis state at the transcriptomic level. PRGScore is defined as:


$$\text{PRGScore}=\left(\text{PC}1+\text{PC}2\right)\times \sum {\text{exp}}_{i}$$

where exp*_i_* is the expression level of pyroptosis-related genes. PC1 and PC2 are the first two principal components resulting from PCA.

In the scRNA-seq dataset, PRGScore is defined as the average expression level of pyroptosis-related genes for each single cell. The estimation of PRGScore was implemented by using the function “AddModuleScore” of the R package “Seurat” (42).

### Statistical Analysis

Correlations between variables were explored using Pearson correlation analysis. Continuous variables fitting a normal distribution between binary groups were compared using a *t* test. For comparisons of more than two groups, Kruskal-Wallis tests were used to compare the differences. The cutoff values of each dataset were evaluated based on the association between survival outcome and PRGScore in each separate dataset using the R package survminer. The Kaplan-Meier method was used to generate survival curves for the subgroups in each data set, and the log-rank test was used to determine statistically significant differences. All statistical analyses were implemented using R 4.0.2 (https://www.r-project.org/). *P* values were two-sided. *P* values lower than 0.05 were considered statistically significant.

## Results

### Genomic and Transcriptomic Landscape of Pyroptosis-Related Regulators in Muscle-Invasive Bladder Cancer

A total of 909 MIBC samples with transcriptomic data were obtained from TCGA database and GSE87304, GSE31684, GSE48075 and GSE169455 cohorts. All public MIBC data integrated in this study are documented in **Supplementary Table 1**. From IMvigor210 and GSE176307, we collected 348 and 90 mUC samples, respectively. The MGSP dataset consisting of 121 individuals were also included.

**Figure 1:**
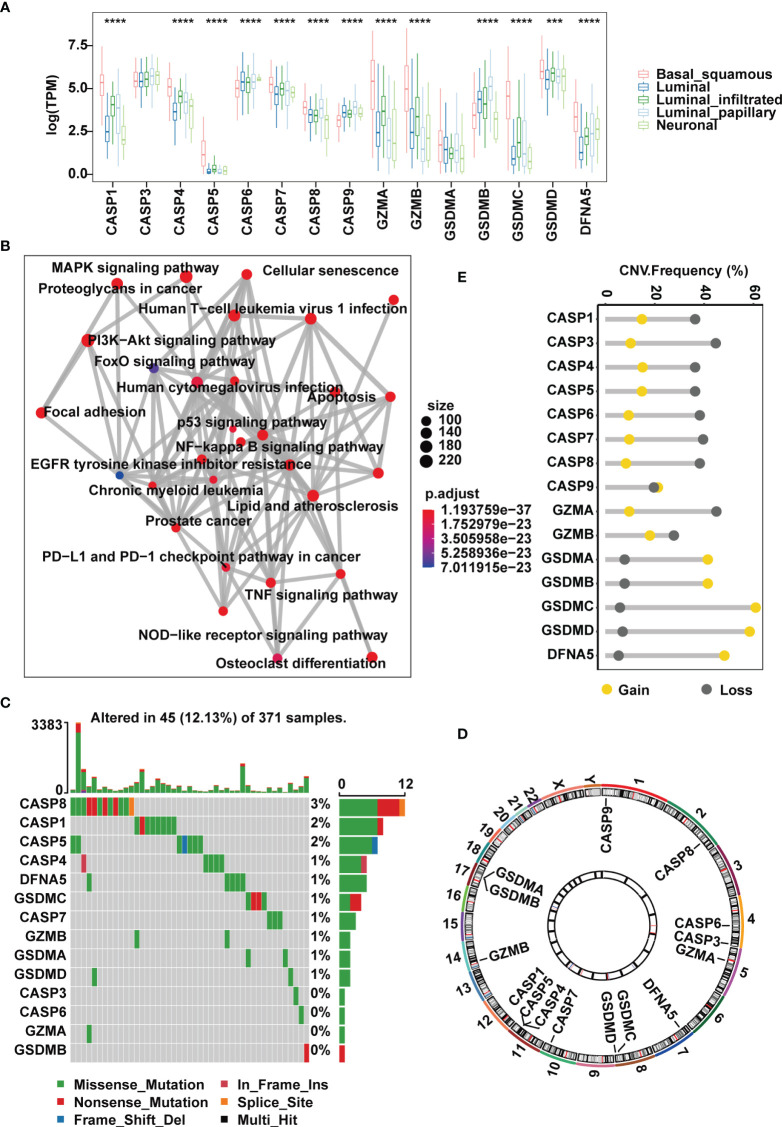
Landscape of genetic and expression variation of pyroptosis regulators in the TCGA-MIBC cohort. **(A)** The expression of 15 pyroptosis regulators in different MIBC molecular subtypes. **(B)** An aggregate of potential biological interactions among pyroptosis-related regulators from a one-step protein-protein interaction network. **(C)** The mutation frequency and classification of 15 pyroptosis regulators in MIBC. **(D)** Genomic position of 15 pyroptosis regulators. Bands at the inner circle indicate corresponding expression levels. **(E)** CNV frequency of 15 pyroptosis regulators. The asterisks represent the statistical P value ( ***p < .001; ****p < 0.0001).

TCGA has completed a comprehensive molecular subtype characterization of bladder cancer and has proposed subdivision of BLCA into five subtypes: luminal infiltrated, luminal papillary, luminal, basal squamous and neuronal. For genes encoding the 10 common pyroptosis-related cleavage enzymes and 5 GSDMs, we first investigated their expression in muscle-invasive samples of the TCGA-MIBC cohort segregated by the five subtypes. We found that the basal squamous subgroup had remarkably higher expression of cleavage enzymes *CASP1, CASP4, CASP5, GZMA* and *GZMB* than the others (**Figure 1A**). However, compared with other subgroups, except for *CASP6*, *CASP7, CASP9* and *DFNA5*, the remaining regulators all showed decreases in neuronal samples (**Figure 1A**). Next, we evaluated potential biological functions associated with these canonical caspases, granzyme proteases and GSDMs in a one-step protein-protein interaction network, which revealed that these regulators were predominantly involved in the regulation of the immune response, such as the NOD-like receptor signaling pathway and PD-L1 and PD-1 checkpoint pathway in cancer, and the regulation of stromal and carcinogenic activation, including TNF signaling pathway, MAPK signaling pathway and p53 signaling pathway (**Figure 1B**).

At the genomic level, 45 (12.13%) of 371 MIBC cases harbored somatic mutations in pyroptosis-related regulators, with *CASP8*, *CASP1* and *CASP5* showing the highest frequency of alterations. However, mutations in *GSDMB*, *GZMA*, *CASP3* and *CASP6* were less common and were found only in individual cases (**Figure 1C**). CNV analysis of the 15 pyroptosis-related regulators suggested that most cleavage enzymes more frequently had copy number deletions, while gasdermins had widespread amplification (**Figures 1D, E**). Of note, we found that *CASP1*, *CASP4* and *CASP5*, which are located within an approximately 50 kb genomic region, shared similar mutation frequencies and similar patterns of CNV, and this was also the case with *GSDMA* and *GSDMB* (**Figures 1C–E**). Together, these evidences indicate that the expression and mutation patterns of pyroptosis-related regulators are highly heterogeneous in MIBC.

### Identification of Pyroptosis Patterns Defined by 10 Canonical Pyroptosis-Related Regulators and *GSDMB* in MIBC

**Figure 2:**
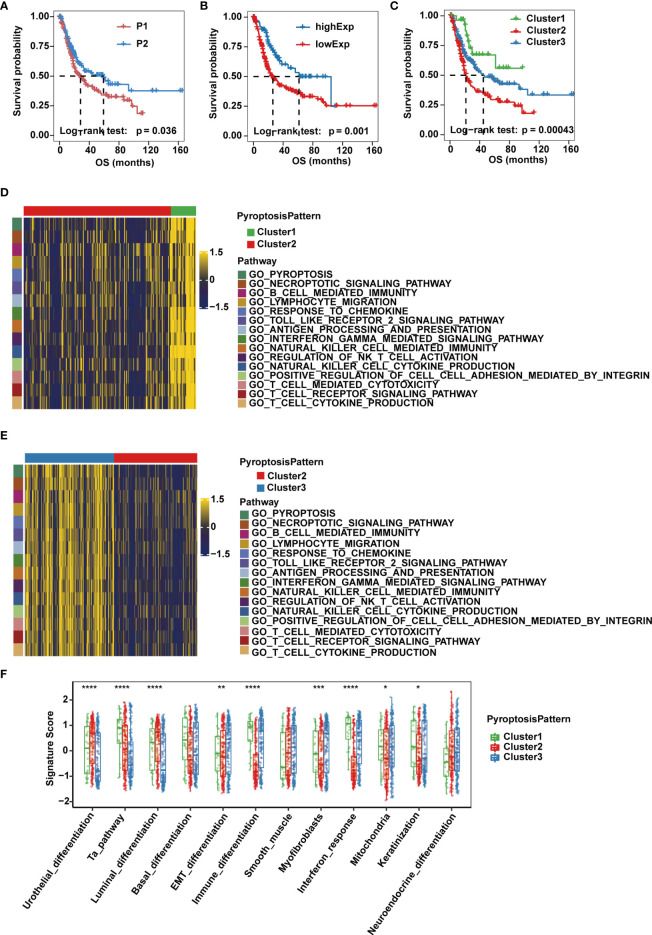
Pyroptosis patterns and biological characteristics of each pattern in the TCGA-MIBC cohort. **(A)** Kaplan-Meier curve for the two cleavage enzyme regulation patterns. **(B)** Survival analysis for patients with high or low *GSDMB* expression using Kaplan-Meier curves. **(C)** Survival analysis of the three pyroptosis patterns based on two cleavage enzyme regulation patterns and *GSDMB* expression. **(D, E)** Heatmaps showing differentially enriched biological pathways in the three pyroptosis patterns by GSVA. **(F)** GSVA enrichment analysis representing the significance of differential expression of specific bladder cancer-related signatures among the three pyroptosis patterns. The asterisks represent the statistical *P* value (*p < 0.05; **p < 0.01; ***p < 0.001; ****p < 0.0001).

Next, we sought to understand how the imbalanced expression and mutation of pyroptosis-related regulators would influence the occurrence and progression of MIBC. A workflow was designed to systemically assess pyroptosis patterns and pyroptosis gene signatures in MIBC (**Supplementary Figure 1A**). Based on consensus clustering of the expression profiles of the 10 pyroptosis-related cleavage enzymes in the TCGA-MIBC cohort, we identified two different cleavage enzyme regulation patterns, namely, pattern P1 (n = 228) and pattern P2 (n = 140) (**Supplementary Figure 1B**). Higher expression levels of *CASP1*, *CASP4*, *CASP5*, *GZMA* and *GZMB* were observed in group P2 (**Supplementary Figure 1C**). In addition, these two regulatory patterns could be further confirmed in the GSE87304 cohort consisting of 305 MIBC cases (**Supplementary Figures 1D, E**).Group P2 also showed a significantly improved overall survival and progression free survival compared to that of group P1 (**Figure 2A** and **Supplementary Figure 2A**).

Given that gasdermins also play an indispensable role in the process of pyroptosis, we then evaluated their prognostic value in MIBC cases of TCGA-BLCA cohort, and found that expression of *GSDMB*, but not *GSDMA*, *GSDMC*, *GSDMD* or *GSDME*, was associated with overall survival, and OS and PFS of *GSDMB*-high group was significantly better than *GSDMB*-low group (**Figure 2B** and **Supplementary Figures 2B, C**). Therefore, we divided patients into three clusters (Clusters 1-3) based on three pyroptosis patterns defined by the two expression patterns of cleavage enzymes and the expression level of *GSDMB*. Cluster 1 consisted of cases with pattern P2 and high *GSDMB* expression, Cluster 2 consisted of cases with pattern P1 and low *GSDMB* expression, and the remaining cases belonged to Cluster 3. Significant differences in OS and PFS were observed for the three clusters, of which Cluster 1 was characterized by the highest OS and PFS, while Cluster 2 had the worst prognosis (**Figure 2C** and **Supplementary Figure 2D**).

### Association Between Pyroptosis Patterns and Clinical and Molecular Characteristics of MIBC

To explore the differences in underlying biological function among the three pyroptosis patterns, we performed GSVA on the three clusters. Clusters 1 and 3 showed enrichment in terms of pathways associated with fully activated immune function, including lymphocyte migration, antigen processing and presentation, TOLL-like receptor signaling pathways, interferon gamma-mediated signaling pathway, B cell receptor signaling pathways, T cell receptor signaling pathways, NK cell-mediated cytotoxicity and chemokine signaling pathway. However, Cluster 2 was prominently associated with biological processes related to immune suppression (**Figures 2D, E**). Next, we quantified pyroptosis activity for the three clusters and found that Cluster 1 was described by pyroptosis activation, Cluster 2 tended to be inactive and disordered in pyroptosis and Cluster 3 had moderate pyroptosis activation (**Supplementary Figure 2E**).

Then, we performed ssGSEA based on the gene expression of individual samples in each dataset to test the significance of the differential expression of specific bladder cancer-related signatures in three distinct clusters (**Supplementary Table 2**). We found that MIBC patients in Cluster 1 were more likely to be enriched in immune differentiation and interferon response (**Figure 2F**). Cluster 2 showed enrichment in terms of pathways associated with urothelial and luminal differentiation but not immune activation. Cluster 3 had medium enrichment in scores of immune differentiation and interferon response and had the highest level of epithelial-mesenchymal transition (EMT) differentiation, myofibroblasts and smooth muscle signature scores (**Figure 2F**). Previous studies reported that although some tumors were found to be rich in immune cells, these immune cells were unable to infiltrate into tumor tissue and were kept in the surrounding matrix. Therefore, stromal activation in the TME is considered to be immunosuppressive due to the formation of an immune exclude phenotype (14). The three pyroptosis patterns were then validated in the GSE87304 dataset, where Cluster 1 was significantly associated with pyroptosis and immune activation, Cluster 2 lacked both pyroptosis and immune activation, and Cluster 3 had intermediate pyroptosis and immune activation but significantly higher stromal activity scores (**Supplementary Figure 2F**). Together, these results provide evidence that the three pyroptosis patterns represent distinct clinical features and are generalized signatures for MIBC.

### Differences in TME Infiltration for the Three Pyroptosis Patterns in MIBC

**Figure 3:**
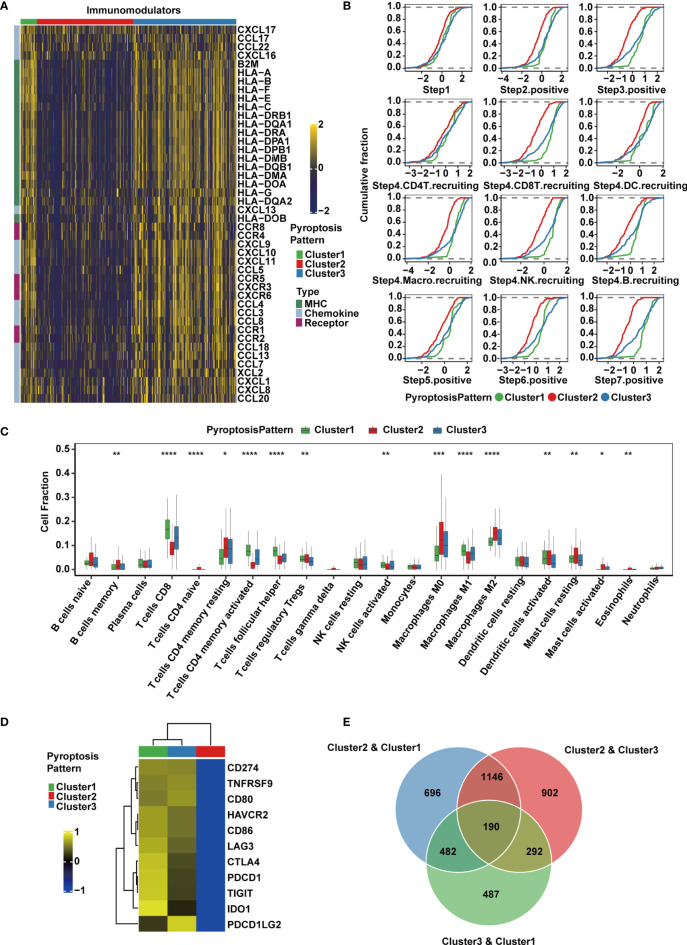
TME and transcriptomic characteristics of the three distinct pyroptosis patterns in the TCGA-MIBC cohort. **(A)** Differences in the expression of chemokines, receptors and MHC molecules between the three pyroptosis patterns in TCGA-MIBC. **(B)** Differences in various steps of the cancer immunity cycle between three pyroptosis patterns in TCGA-MIBC. **(C)** The abundance of each TME-infiltrating cell type in three pyroptosis patterns. The asterisks represent the statistical *P* value (*p < 0.05; **p < 0.01; ***p < 0.001; ****p < 0.0001). **(D)** Heatmap representing the expression level of immune checkpoint genes in the three clusters. **(E)** Venn diagram showing pairwise DEGs for the three pyroptosis patterns.

To explore the immunological characteristics of the TME among these distinct pyroptosis patterns, we estimated the expression of immunomodulators (53) and immune checkpoint genes, activity of the cancer immunity cycle and infiltration level of tumor-infiltrating immune cells. We found that the majority of MHC-I constituents, such as *HLA-A*, *HLA-B*, *HLA-C* and *HLA-E*, and MHC-II components, such as *HLA-DRB1*, *HLA-DQA1*, *HLA-DMB* and *HLA-DRA*, were upregulated in Cluster 1 (**Figure 3A**), indicating an enhancement in the capacity of antigen presentation and processing. Key chemokines and their receptors, including *CCL3*, *CCL4*, *CCL5*, *CXCL9*, *CXCL10*, *CXCL11*, *CXCL13*, *CCR1*, *CCR5* and *CXCR3*, were also significantly upregulated in this group (**Figure 3A**). These chemokines were able to promote the recruitment of CD8^+^ T cells, NK cells, and antigen-presenting cells, which suggested that the TME of Cluster 1 could recruit more antitumor immune cells.

Due to the complex and sophisticated functions and interactions of the chemokine-receptor network, the expression level of individual chemokines was insufficient to clarify the overall immunological activation or exhaustion in the TME. However, by measuring the activities of the cancer immunity cycle, the interactions in the chemokine system and other immunomodulators could be comprehensively summarized (48, 54). Therefore, we set out to explore the activities of cancer immunity cycle in distinct pyroptosis patterns, and found that Cluster1 showed significant upregulation in the majority of the steps in the immunity cycle, including the release of cancer cell antigens (Step 1), cancer antigen presentation (Step 2), priming and activation (Step 3), trafficking of immune cells to tumors such as CD8^+^ T cell, NK cell and B cell recruiting (Step 4), infiltration of immune cells into tumors (Step 5), recognition of cancer cells by T cells (Step 6), and killing of cancer cells (Step 7) (54) (**Figure 3B**). Interestingly, the activities from Step 1 to Step 7 were downregulated in Cluster 2, which may give rise to a reduction in the infiltration level of effector cytotoxic T cells, leading to an anti-inflammatory TME and weaker antitumor effect. The downregulation might be contributed by the immune desert phenotype in Cluster 2. However, cluster 3 had similar levels of cancer immunity cycle activities compared with Cluster 1 except for CD8^+^ T cell recruitment. Thereafter, we used the CIBERSORTx algorithm to deconvolute the infiltration of immune cells in the TME and found that M1 macrophages, along with CD8^+^ T cells, activated NK cells and activated memory CD4^+^ T cells, were abundant in Cluster 1 (**Figure 3C**), implying enhanced antitumor function and a significant survival advantage. Moderate infiltration levels of most immune cells were found in Cluster 3. However, Cluster 2 was enriched with mast cells, M2 macrophages, regulatory CD4^+^ T cells and resting memory CD4^+^ T cells, which suggested that the TME of this cluster presented a status of immunosuppression (**Figure 3C**). In addition, the expression of immunomodulators and immune checkpoint genes, activities of cancer immunity cycle and TME-infiltrating cells in the three patterns were estimated and validated in GSE87304, which further demonstrated that the three pyroptosis patterns were representations of three distinct immune phenotypes including immune inflamed, immune desert and immune excluded and might imply various degrees of antitumor efficacy (**Supplementary Figures 3A**–**C**).

The expression of immune checkpoint inhibitors was reported to be low in the noninflamed TME (55). Consistently, we found that Cluster 1 and Cluster 3 had higher expression levels of a majority of immune checkpoint inhibitory genes, including *CD274* (PD-L1), *PDCD1LG2* (PD-L2), *PDCD1* (PD-1), *CTLA4*, *LAG3*, *HAVCR2* (TIM-3), *IDO1* and *TIGIT*, and they were all downregulated in Cluster 2 in both the TCGA (**Figure 3D**) and GSE87304 (**Supplementary Figure 3D**) cohorts. Together, these findings suggest that the three pyroptosis patterns are significantly different in the cancer immunity cycle and immune cell infiltration in the TME, especially in infiltrating and recruiting CD8^+^ T cells.

### Classification of MIBC Subtypes by Pyroptosis-Related Gene Signatures

**Figure 4:**
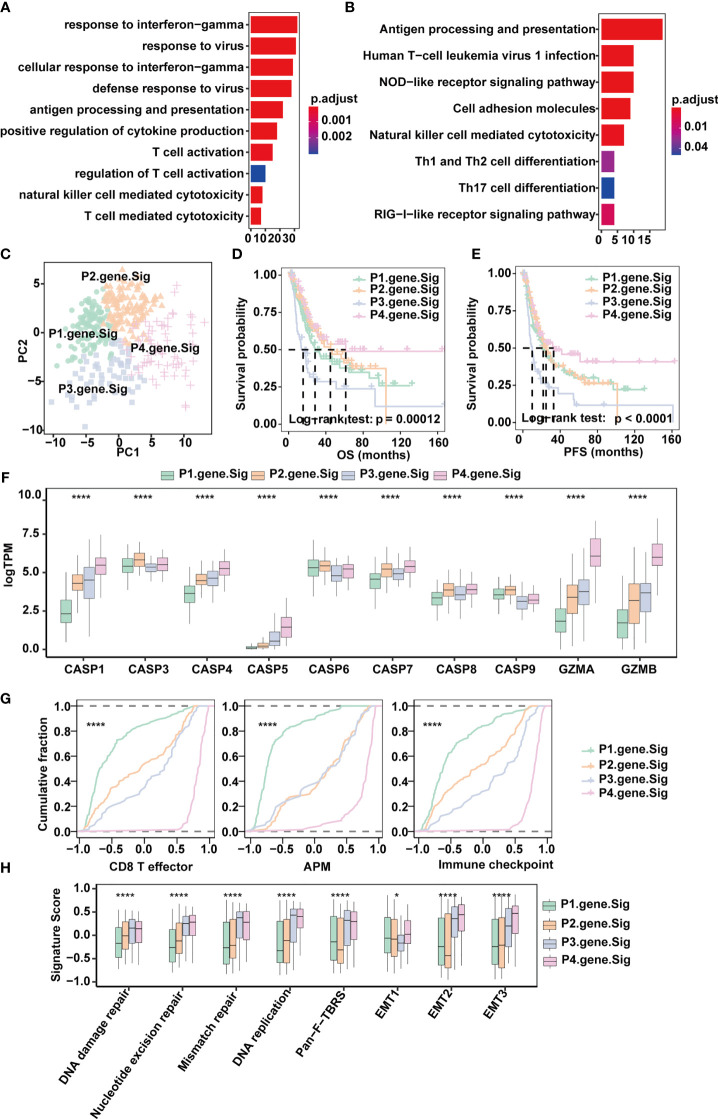
Classification of MIBC subtypes based on pyroptosis signatures in the TCGA-MIBC cohort. **(A)** Enrichment of GO terms and number of genes involved in pyroptosis-related signature genes. **(B)** Enrichment of KEGG pathways and number of genes involved in pyroptosis-related signature genes. **(C)** Principle components built based on the expression of the 57 pyroptosis-related signature genes. **(D)** Overall survival analysis for the four pyroptosis gene signatures. **(E)** Progression-free survival analysis for the four pyroptosis gene signatures. **(F)** Boxplots depicting the differences in pyroptosis enzyme expression between the four pyroptosis gene signatures in the TCGA-MIBC cohort. The asterisks represent the statistical *P* value (*p < 0.05; ****p < 0.0001). **(G)** Cumulative distribution function showing the four pyroptosis gene signatures were distinguished by different signatures (CD8T effector, APM, and Immune checkpoint as indicated) in the TCGA-MIBC cohort. **(H)** Boxplots depicting the four pyroptosis gene signatures were distinguished by different signatures (mismatch-relevant signature and stromal-relevant signature as indicated) in the TCGA-MIBC cohort. The asterisks represent the statistical *P* value (*p < 0.05; ****p < 0.0001).

To quantify the pyroptosis pattern in MIBC, we first identified 190 DEGs across Clusters 1-3 (**Figure 3E**). GO and KEGG enrichment analyses were subsequently performed (**Supplementary Tables 3, 4**). The DEGs showed remarkable enrichment of biological pathways related to immune activation and response, including antigen processing and presentation, T cell activation and NK cell-mediated cytotoxicity (**Figures 4A, B**), which further confirmed that pyroptosis played an indispensable role in the TME of MIBC.

Next, univariate Cox regression was applied for the screening of the 190 DEGs, resulting in 57 candidates that were significantly prognostic (**Supplementary Figure 4A** and **Supplementary Table 5**). Consensus clustering was then performed based on the above 57 DEGs to divide MIBC patients in the TCGA BLCA cohort into four subtypes with distinct expression profiles (pyroptosis-related gene signatures P1-4) (**Figures 4C**–**E**). Patients of the four subtypes experienced different clinical outcomes, and the OS and PFS of P4 was significantly better than the OS and PFS of other subtypes (**Figures 4D, E**). Of note, P4 also showed increased expression of *CASP1*, *CASP4*, *CASP5*, *GZMA* and *GZMB* (**Figure 4F**) and scored highest in CD8^+^ T effector, antigen processing machinery and immune checkpoints gene sets (**Figures 4G, H** and **Supplementary Table 6**). Consistent with the TCGA-MIBC cohort, we also identified four subtypes based on consensus clustering of the GSE87304 cohort with 57 DEGs (**Supplementary Figures 4B, C**), and P4 had higher expression of *CASP1*, *CASP4*, *CASP5*, *GZMA* and *GZMB* and higher enrichment scores of CD8^+^ T effectors, antigen processing machinery and immune checkpoints (**Supplementary Figures 4D, E**). This indicates that pyroptosis-related gene signatures are of good classification efficacy and that P4 was significantly associated with pyroptosis and immune activation in the TME of MIBC.

### PRGScore Quantifies Both the Tumor Microenvironment and Pyroptosis State in MIBC

**Figure 5:**
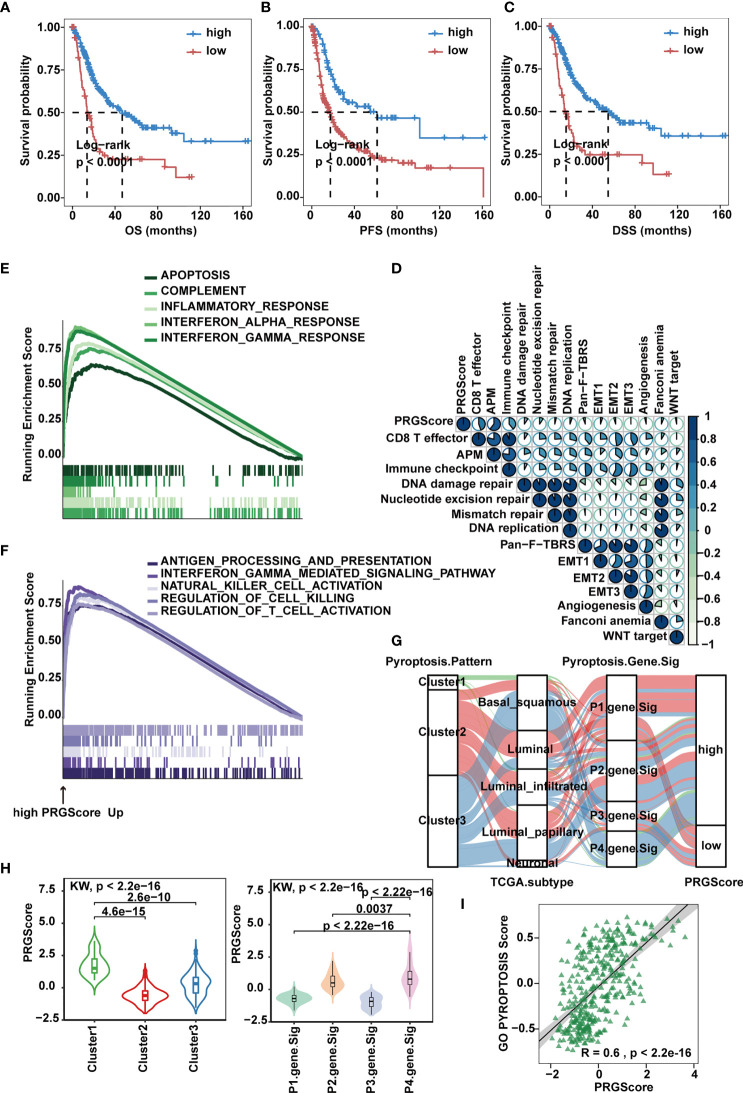
Quantification of pyroptosis signatures based on PRGScore in the TCGA-MIBC cohort. **(A–C)** Survival analysis for overall survival (OS) **(A)**, progression-free survival (PFS) **(B)** and disease-specific survival (DSS) **(C)** for patients with high and low PRGScores in the TCGA-MIBC cohort. **(D)** Correlations between PRGScore and gene signatures linked to EMT, immune checkpoint, mismatch repair, and immune activation in the TCGA-MIBC cohort. **(E)** GSEA identified apoptosis, complement, inflammatory response, interferon α response and interferon λ response enriched in the high PRGScore group based on the HALLMARK pathway. **(F)** GSEA identified immune activation-related pathways (such as the interferon gamma-mediated signaling pathway, antigen processing and presentation, natural killer cell activation, regulation of cell killing and regulation of T cell activation) enriched in the high PRGScore group based on the GO pathway. **(G)** Alluvial diagram showing the connection of pyroptosis patterns, TCGA-MIBC molecular subtypes, pyroptosis-related gene signature and PRGScore. **(H)** Differences in PRGScore among three pyroptosis patterns (left) and among four pyroptosis-related gene signatures (right) in the TCGA-MIBC cohort. The Kruskal-Wallis test was used to compare the significant differences between three gene clusters. **(I)** Scatter plots depicting the significantly positive correlation between PRGScore and GO pyroptosis signature score in the TCGA-MIBC cohort.

To make these MIBC subtypes defined by pyroptosis-related gene signatures available to clinical practice, we performed PCA on the 57 DEGs and defined a scoring system named PRGScore to quantify the pyroptosis status for each MIBC case (**Figure 4C**). We found that the PRGScore-high group showed a prominent survival benefit, while the PRGScore-low group exhibited much poorer survival (**Figures 5A**–**C**).

Next, we evaluated the relation between PRGScore and TME features. By applying the GSVA algorithm, we found a significant positive correlation between PRGScore and enrichment scores of CD8^+^ T effectors, antigen processing machinery and immune checkpoints in TCGA-MIBC, GSE87034 and three external GEO datasets (**Figure 5D** and **Supplementary Figure 5A**). Subsequently, we employed GSEA with all transcripts ranked by the log2 (fold-change) between high and low PRGScore group based on Hallmark and GO pathway, and found enrichment in gene sets related to immune activation in high PRGScore group, including complement, inflammatory, interferon alpha and gamma mediated signaling pathway, antigen processing and presentation, natural killer cell activation, and regulation of T cell activation (**Figures 5E, F**).

An alluvial diagram was used to visualize the attribute changes of individual patients. Consistent with the above findings, Cluster 2 with the neuronal subtype (TCGA molecular subtypes) was linked to a low PRGScore (**Figure 5G**). The Kruskal-Wallis test revealed significant differences in PRGScore among distinct pyroptosis clusters. While Cluster 2 scored the lowest, Cluster 1, associated with pyroptosis activation, had a significantly increased PRGScore compared to the others (**Figure 5H** and **Supplementary Figure 5B**). More importantly, pyroptosis-related gene signature P4 showed the highest median PRGScore compared to the other clusters, while P1 and P3 had low PRGScore (**Figure 5H** and **Supplementary Figure 5B**). Interestingly, PRGScore patterns for Clusters 1-3 showed high consistency with corresponding GO biological process pyroptosis scores (**Figure 5I** and **Supplementary Figure 5C**), suggesting a strong link between these two measurements. Together, these findings indicate that a high PRGScore is closely linked to enhanced pyroptotic cell death and immune activation signatures. To further test its stability, we applied the PRGScore established in the TCGA-MIBC cohort to other independent MIBC cohorts, including GSE169455, GSE48075 and GSE31684, and again it showed good performance in predicting prognosis (**Supplementary Figures 5D, E**). We then analyzed the correlation between PRGScore and survival rate by multivariate Cox regression analysis and proved that PRGScore was an independent and robust prognostic factor for MIBC (**Supplementary Figure 5F**).

### High PRGScore Implies an Immune-Active Tumor Microenvironment

**Figure 6:**
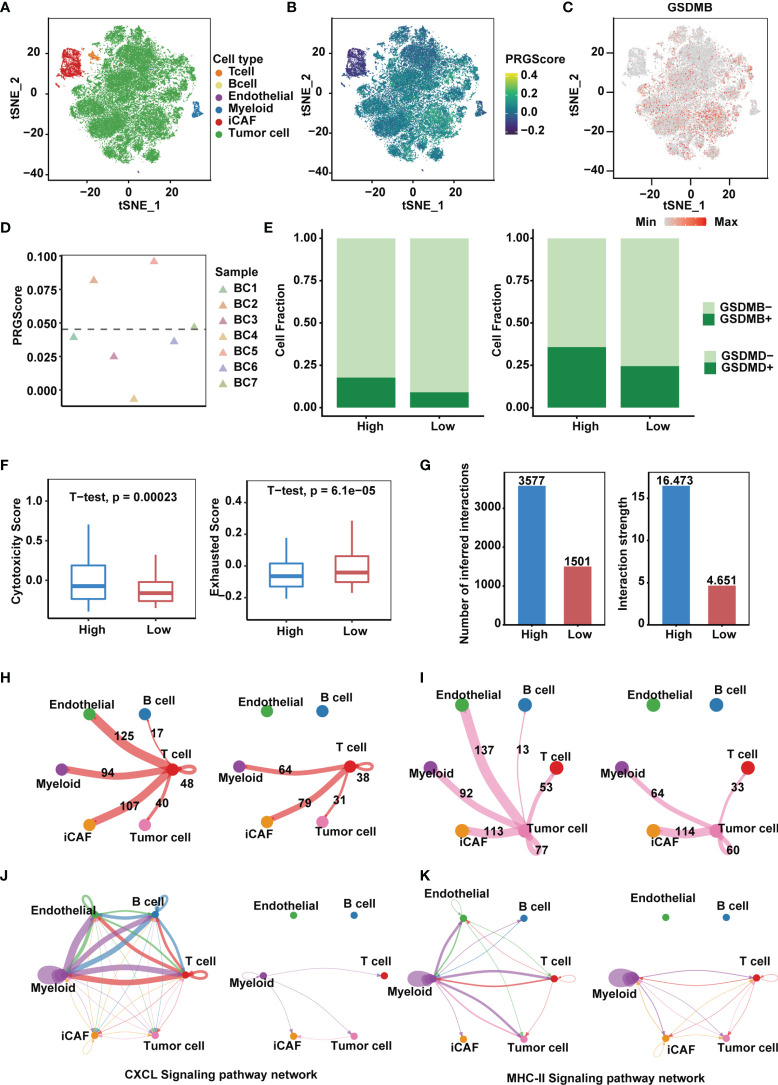
High PRGScore implies an immune-active tumor microenvironment in the single cell cohort. **(A)** t-SNE plot showing the composition of 6 main subtypes derived from bladder cancer samples. **(B)** The dynamics of PRGScore in 6 main cell types are represented in the t-SNE plot. **(C)** t-SNE plot showing the expression level of *GSDMB* for all the cell types. **(D)** Distribution in the PRGScore of seven bladder cancer samples (divided into 2 patterns). **(E)** The proportion of *GSDMB* ^+^ tumor cells (left) and *GSDMD* ^+^ tumor cells (right) in the high or low PRGScore group. **(F)** Differences in cytotoxic score (left) and exhausted score (right) of T cells between two pyroptosis groups. **(G)** Differences in number of inferred interactions (left) and interaction strength (right) of all cells between two pyroptosis groups. **(H)** Circos plots displaying putative ligand-receptor interactions between T cells and other cell clusters from high-PRGScore (left) and low-PRGScore (right) group. The brand links pairs of interacting cell types, and corresponding number of events were labeled in the graph. **(I)** Circos plots displaying putative ligand-receptor interactions between Tumor cells and other cell clusters from high-PRGScore (left) and low-PRGScore (right) group. The brand links pairs of interacting cell types, and corresponding number of events were labeled in the graph. **(J)** Circos plots showing the CXCL signaling pathways between high-PRGScore (left) and low-PRGScore (right) group. **(K)** Circos plots showing the MHC-II signaling pathways between high-PRGScore (left) and low-PRGScore (right) group.

We next sought to identify key players in the TME that contribute to pyroptotic phenotypes. Single-cell mRNA profiles of seven primary tumor and one normal tissue sample from the GSE135337 dataset were obtained. After quality control and removal of batch effects, filtered cells were clustered and annotated into 6 major clusters, including epithelial (tumor) cells, endothelial cells, inflammatory cancer-associated fibroblasts (iCAFs), T cells, myeloid cells and B cells (**Figures 6A, B** and **Supplementary Figure 6A**). Compared with other GSDMs, remarkable *GSDMB* expression was observed in bladder cancer cells (**Figure 6C** and **Supplementary Figure 6B**), based on which we divided 7 tumor samples into high and low PRGScore groups (**Figure 6D** and **Supplementary Figure 6C**). The proportion of *GSDMB* ^+^ and *GSDMD* ^+^ malignant cells was also higher in the PRGScore-high group compared with low PRGScore group (**Figure 6E**). Consistent with previous results, we observed higher cytotoxic scores and lower exhausted scores of the T cells for samples with high PRGScore (**Figure 6F**). To characterize intercellular interactions in high and low PRGScore group, we inferred putative cell-to-cell interactions based on ligand-receptor signaling using CellChat. Interestingly, we observed enhanced intercellular interactions for the high PRGScore group (**Figure 6G**), where T cells, tumor cells and myeloid cells displayed widespread communication with other cell types, indicating that they were potential contributors to the pyroptotic phenotype (**Figures 6H, I** and **Supplementary Figure 6D**). In addition, we found that CXCL, MHC-II and CCL signaling networks were strengthened, suggesting that they play a crucial role in the progression of MIBC (**Figures 6J, K** and **Supplementary Figure 6E**). We then dissected the signaling networks to identify individual ligand-receptor pairs that were featured in the PRGScore high samples. Enhanced signaling from T cells to tumor cells and myeloid cells was observed in the PRGScore high group, including CSF3-CSF3R, which might mediate the maintenance and proliferation of macrophages (**Supplementary Figure 6F**). Besides, we found increased communication probability between T cells and myeloid cells *via* CCL3-CCR5, CCL5-CCR5, CCL5-CCR1, CCL4-CCR5 and CXCL12-CXCR4, suggesting the enhanced recruitment of T cells by macrophages in the PRGScore high group. Moreover, interactions related to interferon mediated signaling pathway and antigen processing and presentation (IFNG-IFNGR1, IFNG-IFNGR2, HLA-F-CD8A, HLA-DRB5-CD4, HLA-DRA-CD4, HLA-DQB1-CD4 and HLA-DQA1-CD4) were also up-regulated in the PRGScore high group (**Supplementary Figure 6F**). Collectively, these results confirmed the positive relation between the pyroptotic phenotype and the immune activity.

### PRGScore Predicts Clinical Response to Immune Checkpoint Inhibitor Therapy in MIBC

**Figure 7:**
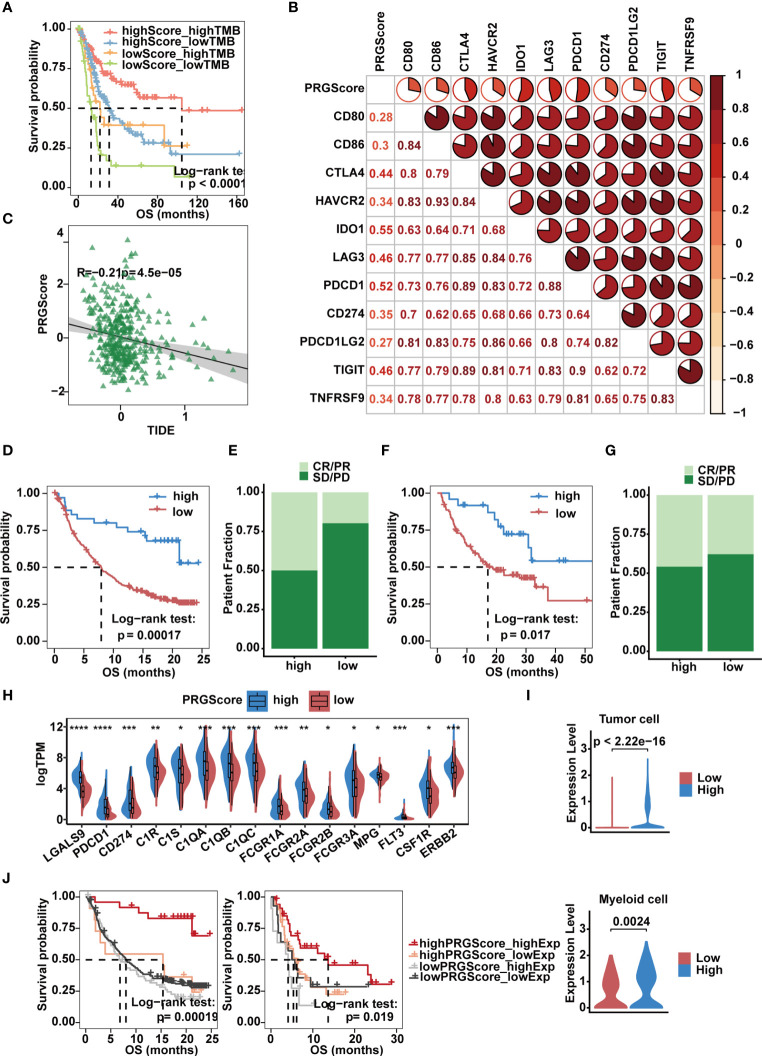
Implication of the PRGScore and its role in the prediction of response to anti-PD-1/L1 immunotherapy. **(A)** Kaplan-Meier curves showing that PRGScore was significantly associated with favorable survival outcome in either the high or low TMB subset of the TCGA-MIBC cohort. **(B)** Correlations between PRGScore and immune checkpoint gene expression in the TCGA-MIBC cohort. **(C)** Scatter plots showing the significantly negative correlation between PRGScore and TIDE score in the TCGA-MIBC cohort. The Pearson correlation between PRGScore and TIDE score is shown. **(D)** Survival analysis for patients with high and low PRGScores in the IMvigor210 cohort. **(E)** The proportion of patients with or without response to PD-L1 blockade therapy in the PRGScore-high and PRGScore-low groups in the IMvigor210 cohort. **(F)** Kaplan-Meier curve showing OS for the PRGScore-high and PRGScore-low groups in the melanoma cohort. **(G)** The proportion of patients who responded to immune checkpoint blockade therapy in the PRGScore-high and PRGScore-low groups in the melanoma cohort. **(H)** Expression of BLCA-related drug targets in the PRGScore-high and PRGScore-low groups of the TCGA-MIBC cohort. The asterisks represent the statistical *P* value (*p < 0.05; **p < 0.01; ***p < 0.001; ****p < 0.0001). **(I)** Expression of *LGALS9* in the PRGScore-high and PRGScore-low groups of the tumor cells (top) and myeloid cells (bottom) in the single cell dataset. **(J)** Kaplan-Meier curves showing that PRGScore was significantly associated with favorable survival outcome in either the high or low *LGALS9* expression subset of the IMvigor210 and GSE176307 cohorts.

Immunologic checkpoint inhibitors (ICIs) that block the T cell inhibitory molecules PD-1 and PD-L1 have undoubtedly emerged as a famous anticancer treatment with unprecedentedly improved survival benefits (56). Previous studies suggest that the level of TMB can reflect the potential of immunogenicity and correlates with the response to immune checkpoint inhibitors (14). Given that MIBC is characterized by one of the highest somatic mutation rates (57), by combining PRGScore with TMB, we witnessed an improvement in the survival prediction with the TCGA-MIBC cohort. In brief, patients with high PRGScore and TMB showed better prognosis, patients with low PRGScore and TMB experienced much poorer prognosis, and those in the other two groups exhibited intermediate prognosis (**Figure 7A**). Therefore, the positive correlation between TMB and PRGScore served as evidence that pyroptosis status could be a crucial factor influencing the clinical response of MIBC to anti-PD-1/PD-L1 immunotherapy.

By investigating the relationship between PRGScore and gene expression, we found that PRGScore positively correlated with a majority of immune checkpoint genes, which indicated a pharmacologic effect of anti-PD-1/L1 immunotherapy on patients with high PRGScores inTCGA-MIBC and other GEO datasets (**Figure 7B** and **Supplementary Figure 7A**). TIDE is a computational framework for modeling the induction of T cell dysfunction in tumors with high infiltration of cytotoxic T lymphocytes and the prevention of T cell infiltration in tumors with low cytotoxic T lymphocyte infiltration levels (58). The TIDE score is significantly correlated with the ICI therapy response rate. Therefore, we explored whether PRGScore could be used to evaluate the responses of ICI therapy in TCGA-MIBC and other GEO cohorts by applying the TIDE algorithm to estimate ICI therapy efficacy. As a result, we found a significantly negative correlation between PRGScore and TIDE score in these datasets (**Figure 7C** and **Supplementary Figure 7B**).

To further investigate whether PRGScore could predict patients’ response to ICI therapy in the immunotherapy cohort, we next explored the prognostic value of PRGScore on patients who received ICI therapy in the IMvigor210, GSE176307 and metastatic melanoma immunotherapy cohorts by dividing them into PRGScore-high and PRGScore-low groups. Patients with a high PRGScore had significantly longer overall survival than those with a lower PRGScore in both the IMvigor210 cohort (**Figures 7D, E**) and metastatic melanoma cohort (**Figures 7F, G**). Subsequently, we also found that three immune phenotypes, immune desert, immune excluded and immune inflamed, represented different levels of PRGScore (**Supplementary Figure 7C**), which suggested that a higher PRGScore represented a status of immune-inflamed TME. A previous study showed that PD-L1 expression on immune cells is associated with the response of metastatic urothelial cancer to the anti-PD-L1 agent atezolizumab (14). By evaluating the PRGScore in the IMvigor210 cohort that received atezolizumab treatment, we found that the PRGScore was significantly higher in patients with upregulated PD-L1 expression on immune cells (**Supplementary Figure 7D**). Moreover, positive correlations between PRGScore, TMB, and tumor neoantigen burden (TNB) were also observed in the IMvigor210 cohort (**Supplementary Figure 7E**), which indicated a significant association between PRGScore and favorable responses to atezolizumab treatment. Furthermore, patients in the PRGScore-high group showed a remarkable advantage in overall survival (OS) and progression-free survival (PFS) compared with the PRGScore-low group, which could be validated in the GSE176307 cohort (**Supplementary Figures 7F, G**). Together, these findings suggest that the PRGScore is a novel indicator of the response of MIBC to ICI therapy.

### PRGScore Defines a MIBC Subtype Sensitive to Immune Checkpoint Blockade Therapy, Neoadjuvant and Adjuvant Chemotherapy

The molecular subtype of a tumor can predict its clinical response to neoadjuvant chemotherapy, radiotherapy, and several targeted therapies (8, 59). To screen out potential small-molecule compounds for the treatment of MIBC, we further analyzed the expression of drug target genes in MIBC and observed a significant difference between the PRGScore low and high groups (**Supplementary Table 7**). This difference indicated a significantly higher response to nivolumab (*PDCD1*), atezolizumab (*CD274*), avelumab (*CD274*), durvalumab (*CD274*), cetuximab (*C1R*, *C1S*, *C1QA*, *C1QB*, *C1QC*, *FCGR1A*, *FCGR2A*, *FCGR2B* and *FCGR3A*), cisplatin (*MPG*), Trastuzumab (*ERBB2*) and sunitinib (*CSF1R*) in the PRGScore-high group (**Figure 7H**). However, remarkable *LGALS9* expression was observed in patients with a high PRGScore (**Figure 7H**). And we also found that tumor and myeloid cells with stronger expression of *LGALS9* in the high PRGScore group in the single cell dataset (**Figure 7I**). Moreover, we confirmed that patients with activate pyroptotic state and higher *LGALS9* exprssion had improved overall survival rate in the IMvigor210 and GSE176307 cohorts, suggesting that these patients with activate pyroptotic state were more sensitive to the *LGALS9* inhibitors (**Figure 7J**). These results showed that immune checkpoint blockade therapy and neoadjuvant or adjuvant chemotherapy can be used, either alone or in combination, for the treatment of MIBC patients with a high PRGScore.

## Discussion

Bladder cancer is a common malignancy of the urinary system, and MIBC is a more aggressive disease state. MIBC is characterized by poor prognosis and lacks effective therapeutic options (60). Recent reports have shown that pyroptosis is a fulminant form of monocyte and macrophage cell death, contributing to the release of proinflammatory cytokines (61, 62), and PD-L1-mediated *GSDMC* expression switches apoptosis to pyroptosis in cancer cells and facilitates tumor necrosis (63). Several studies indicated that pyroptosis-related modulators are tumor-suppressive in colorectal cancer (64), liver cancer (63) and skin cancer (65), but they exert a dual function in breast cancer (66). However, the role of pyroptosis in MIBC has not been elucidated. Here, we comprehensively characterized pyroptosis-related clinical and molecular features in MIBC by an integrated analysis of public datasets. By quantifying the expression of *GSDMB* and 10 canonical pyroptosis-related cleavage enzymes in the TCGA-MIBC cohort, we identified 3 pyroptosis patterns in MIBC, including pyroptosis activation (Cluster 1), pyroptosis inactivation (Cluster 2) and moderate pyroptosis activation (Cluster 3), which were significantly associated with prognosis and TME infiltration. Based on DEGs of the pyroptosis patterns, we classified MIBC and defined 4 pyroptosis-related MIBC subtypes that experienced distinct clinical outcomes. A scoring system, PRGScore, was designed to comprehensively quantify the pyroptosis state of individual MIBC cases. High PRGScore was significantly correlated with high TMB and increased enrichment of CD8^+^ T effectors in the TME, while low PRGScore was associated with metastasis and poor clinical outcomes.

The inhibition of immunoinhibitory molecules such as PD-1 and PD-L1 can lead to tumor regression by restoring the cytotoxicity of immune cells (67). To date, several ICIs, such as atezolizumab (PD-L1 inhibitor) and nivolumab (PD-1 inhibitor), have been approved by the Food and Drug Administration (FDA) for the treatment of advanced MIBC (68, 69), yet the responses of patients to ICI therapy vary greatly, with some patients achieving complete remission and others showing continuous progression (70). Here, we showed that the PRGScore was significantly associated with the response of MIBC to ICI therapy and that a high PRGScore implied increased sensitivity to ICI, neoadjuvant and adjuvant chemotherapy, which suggested that the application of the PRGScore could assist in decision making for the treatment of MIBC.

Apart from immunotherapy, targeted is becoming the foundation of precision medicine. A recent study showed that the combination of PD-1 inhibitor and induction of target cell pyroptosis effectively inhibits tumor cell proliferation in the mouse colon carcinoma cell line CT26 (25). Our study revealed that *LGALS9* was up-regulated in high PRGScore group compared to low group in the bulk and single cell datasets and enhanced interactions involving LGALS9-HAVCR2, LGALS9-CD45 and LGALS9-CD44 in MIBC with high PRGScores were observed in the single cell dataset. Galectin-9 encoded by *LGALS9* is a tandem protein which contains two ligand-binding domains fused together by a peptide linker (71). Galectin-9 was reported previously to bind with TIM-3 to induce T-helper type 1 lymphocyte (Th1) death (72) and the interaction between Galectin-9 and CD44 enhances the binding of *SMAD3* to the *FOXP3* promoter, leading to up-regulation of *FOXP3* expression and increased induced regulatory T (iTreg) cell stability and suppressive function (73). Galectin-9 ligation also down-regulates multiple immune-activating genes, including eight involving NK cell-mediated cytotoxicity, and reduces the proportion of gamma interferon (IFN-γ)-producing NK cells (74). Moreover, recent study suggests that Galectin-9 interacts with PD-1 and TIM-3 to regulate T cell death, making it a promising target for cancer immunotherapy (75). Because *GSDMB* were preferentially expressed by tumor cells of MIBC, targeted induction of tumor cell pyroptosis could be theoretically achieved. Our study implied that the combination of PD-1 and LGALS9 inhibition and induction of target cell pyroptosis could possibly inhibit tumor proliferation and improve patient survival of MIBC.

As indicated by the concept of design, the PRGScore has a positive correlation with the cellular pyroptotic state. However, of the 15 pyroptosis-related regulators, only *GSDMB* was included in the 57 overlapping DEGs that were used as the basis of PRGScore. As a result, pyroptosis was not listed as a significantly enriched pathway in the GSEA of the 57 DEGs. However, this is still explainable because the remaining 56 DEGs might exhibit more noticeable changes in their expression than the other pyroptosis-related regulators. An example is *forkhead box protein A1* (*FOXA1*), which belongs to the *FOX* gene family. *FOXA1* is reported to be an oncogene in a variety of cancers, including thyroid cancer (76), lung cancer (77), oesophageal cancer (77), and prostate cancer (78). Of note, we found a strong connection between PRGScore and TME features. This could be explained by the existence of several immune-related genes in the DEG list, such as *TNFRSF14* and *HLA*. Although the PRGScore alone serves as an effective predictor for the prognosis and clinical response in MIBC, the roles that some of the founding members of the PRGScore play in MIBC remain largely unknown. Therefore, characterization of these genes might provide more insights into this aggressive urothelial carcinoma.

In brief, our analysis indicates that the PRGScore is an independent risk factor for MIBC, thereby providing an ideal predictor for the prognosis and therapeutic response of MIBC patients. One limitation of this study is that the stability of the PRGScore was tested and validated in a limited number of 7 independent cohorts and 1 scRNA-seq dataset. To prove the reliability of the pyroptosis-related gene signature, studies involving prospective cohorts are needed. In addition, scRNA-seq, a state-of-the-art technology, should be further integrated for future analysis to address possible differences in tumor heterogeneity, immune cell infiltration and intercellular communication between PRGScore-high and PRGScore-low groups at single-cell resolution. Moreover, both *in vitro* and *in vivo* experiments should be conducted on the discovered DEGs for an in-depth characterization of the mechanisms underlying pyroptotic regulation and the progression of MIBC in the future.

## Data Availability Statement

Publicly available datasets were analyzed in this study. This data can be found here: TCGA data at https://portal.gdc.cancer.gov/ and http://xena.ucsc.edu/, GSE31684, GSE48075, GSE87304, GSE176307, GSE169455 and GSE135337 at https://www.ncbi.nlm.nih.gov/geo/, MGSP at https://www.nature.com/articles/s41591-019-0654-5, IMvigor210 at http://research-pub.gene.com/IMvigor210CoreBiologies.

## Author Contributions

WC and QZ conceived and designed the project. QZ, YT, and JZ drafted the manuscript with input from all authors. QZ, YT, JZ, YS, JQ, and DZ revised the manuscript. QZ collected the data and conducted bioinformatics analysis. YT, JZ, YS, JQ, and DZ provided analytical and technical support. QZ and YT participated in the production of figures. All authors contributed to the article and approved the submitted version.

## Funding

This work was supported by the National R&D Program of China (2018YFC2000100, 2019YFA0110900 to WC), the CAS Strategic Priority Research Program (XDA16010102 to WC), the National Natural Science Foundation of China (82173061 to WC).

## Conflict of Interest

The authors declare that the research was conducted in the absence of any commercial or financial relationships that could be construed as a potential conflict of interest.

## Publisher’s Note

All claims expressed in this article are solely those of the authors and do not necessarily represent those of their affiliated organizations, or those of the publisher, the editors and the reviewers. Any product that may be evaluated in this article, or claim that may be made by its manufacturer, is not guaranteed or endorsed by the publisher.
