# Supplementary material for: Pyroptosis-Related Signature Predicts Prognosis and Immunotherapy Efficacy in Muscle-Invasive Bladder Cancer
Source: Front Immunol. 2022 Apr 11;13:782982. doi: 10.3389/fimmu.2022.782982 (PMC9035667; doi:10.3389/fimmu.2022.782982)
Supplement: Supplementary file 1 [file DataSheet_1.pdf]

SUPPLEMENTARY MATERIAL

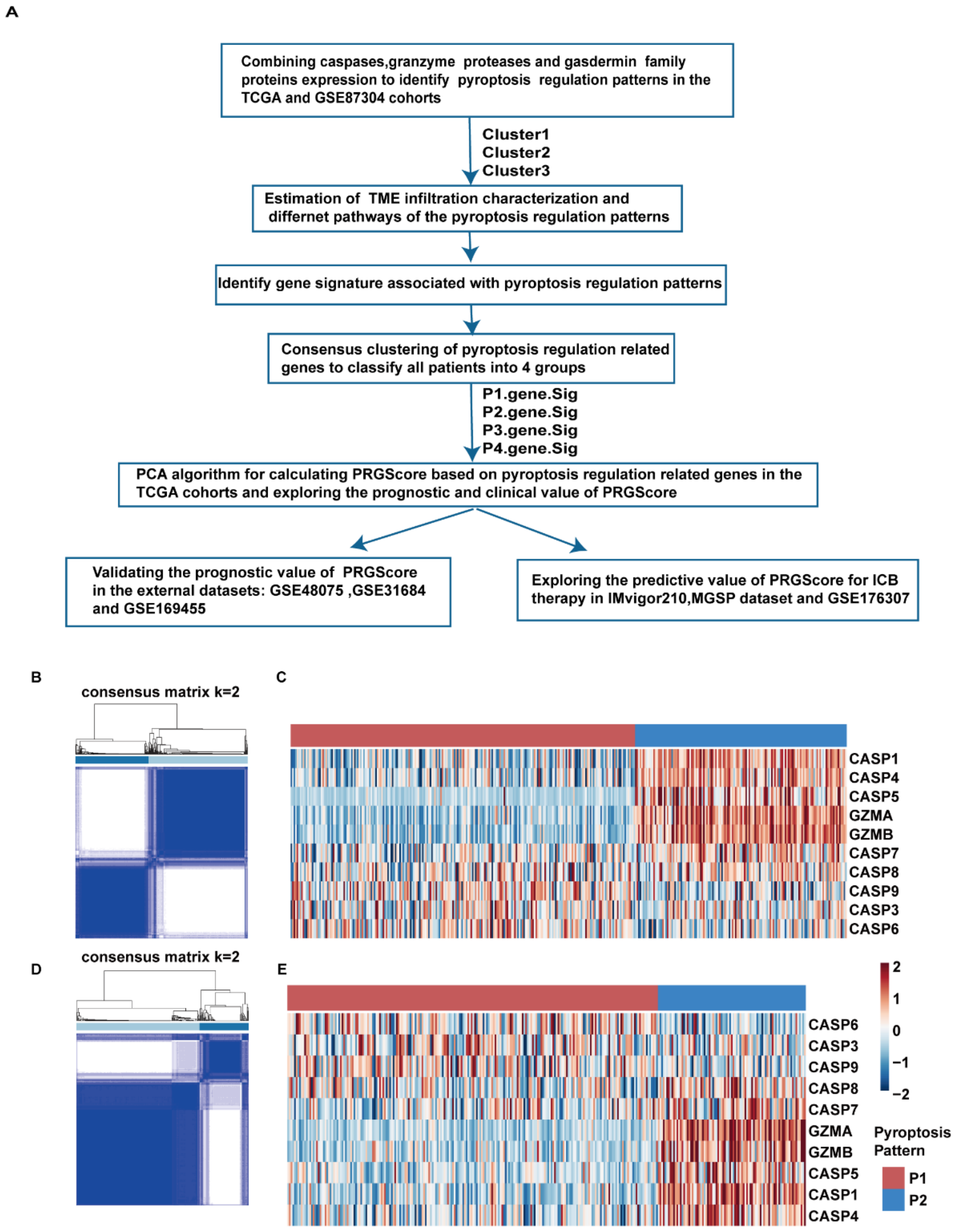

Supplementary Figure 1 | **(A)** Workflow overview. **(B)**The consensus score matrix of all samples when k = 2 in the TCGA-MIBC cohort using 1000 iterations of the K-Means algorithm. **(C)** Consensus clustering of gene expression of cleavage enzymes of TCGA-MIBC cohort. **(D)** The consensus score matrix of all samples when k = 2 in the GSE87304 cohort using 1000 iterations of the K-Means algorithm. **(E)** Consensus clustering of gene expression of cleavage enzymes of GSE87304 cohort.

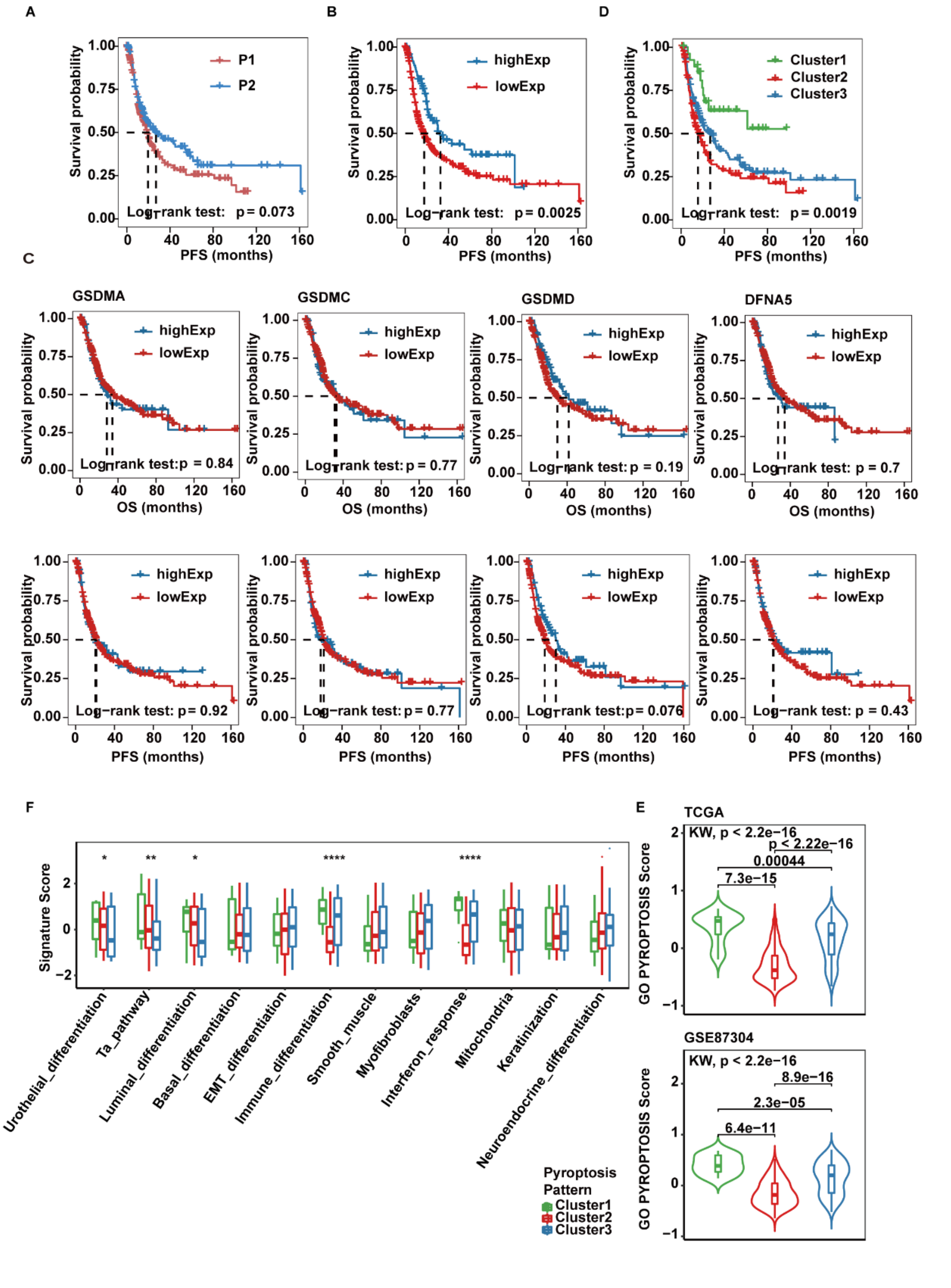

Supplementary Figure 2 | (A) Progression free survival analysis for the two cleavage enzyme regulation patterns. **(B)** Progression free survival analysis for patients with high or low *GSDMB* expression using Kaplan-Meier curves. **(C)** Overall survival (top) and progression free survival (bottom) analysis for patients with high or low *GSDMA*, *GSDMC*, *GSDMD* and *DFNA5* expression in the TCGA-MIBC cohort using Kaplan-Meier curves. **(D)** Progression free survival analysis of the three pyroptosis

patterns based on two cleavage enzyme regulation patterns and *GSDMB* expression.**(E)** Distribution of GO pyroptosis pathway activities among three clusters in TCGA-MIBC (top) and GSE87304 (bottom) cohorts. The differences among groups were compared through the Kruskal-Wails test. *P* values are indicated. **(F)** GSVA enrichment analysis representing the significance of differential expression of specific bladder cancer-related signatures among distinct pyroptosis activation patterns in GSE87304. The asterisks represent the statistical *P* value (* $p<0.05$; ** $p<0.01$; *** $p<0.001$; **** $p<0.0001$).

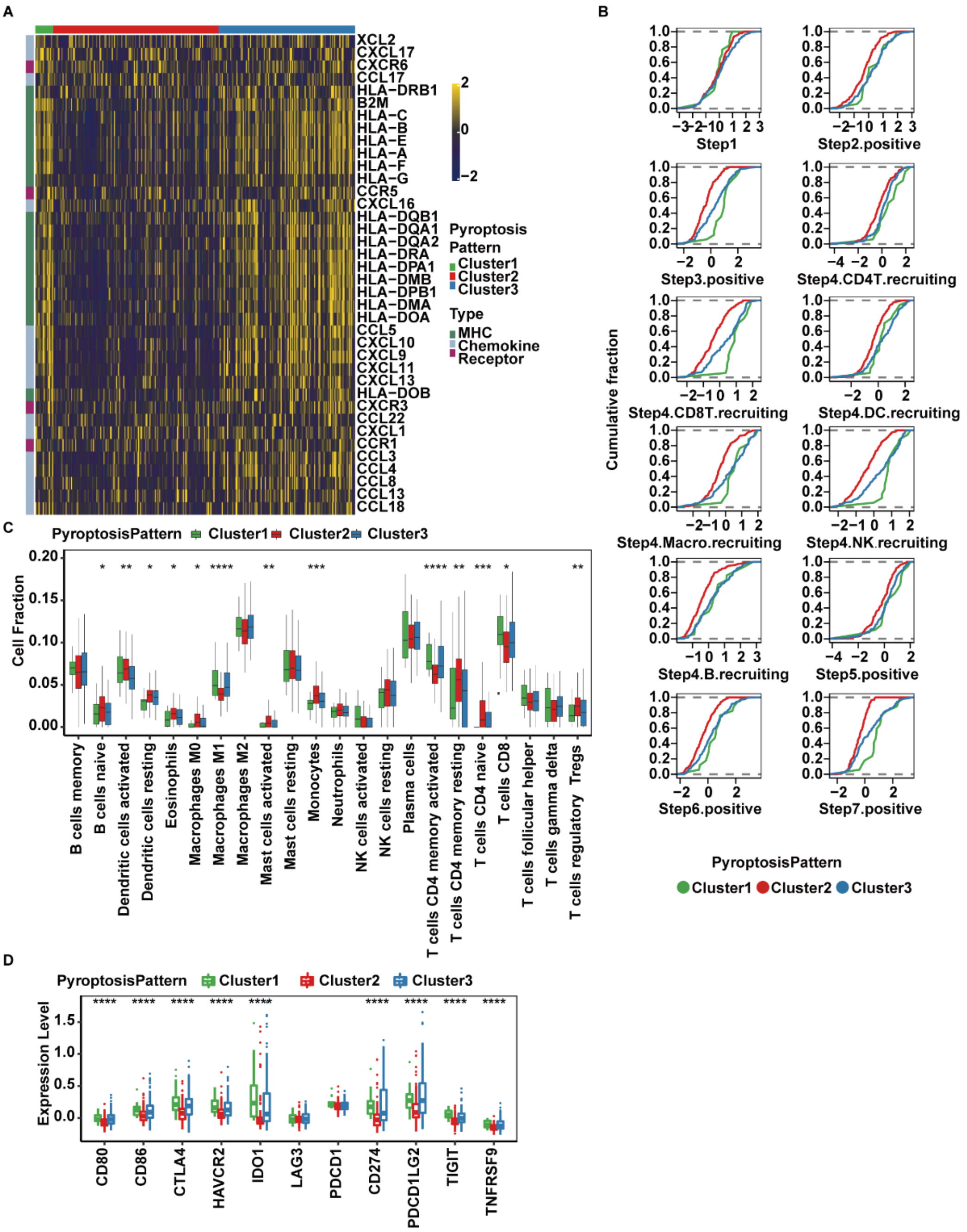

Supplementary Figure 3 | (A) Differences in the expression of chemokines, receptors and MHC molecules between three pyroptosis patterns in the GSE87304 dataset. **(B)** Differences in the various steps of the cancer immunity cycle for the three pyroptosis patterns in the GSE87304 dataset. **(C)** The abundance of each TME infiltrating cell in three pyroptosis patterns in the GSE87304 dataset. The asterisks represent the statistical *P* value (* p<0.05; ** p<0.01; *** p<0.001; **** p<0.0001). **(D)** Boxplots showing the expression levels of immune checkpoint genes in different pyroptosis patterns. The asterisks represent the statistical *P* value (* p<0.05; ** p<0.01; *** p<0.001; ****

$p<0.0001$).

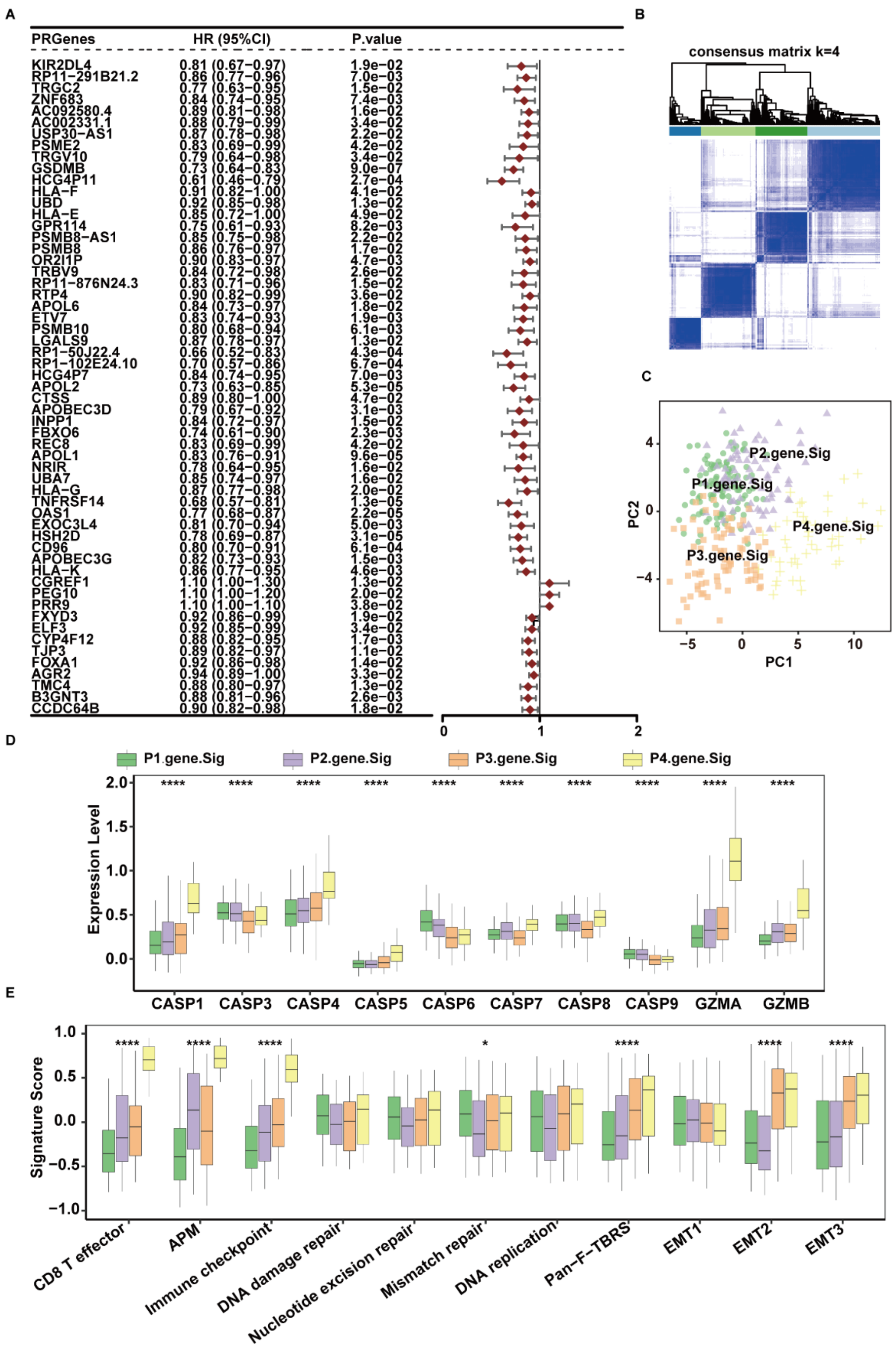

Supplementary Figure 4 | **(A)** Hazard ratio and *P* value for each pyroptosis-related signature gene by applying univariate Cox regression in TCGA-MIBC. **(B)** The consensus score matrix of all samples when k = 4 in the GSE87304 cohort using 1000 iterations of the K-Means algorithm based on pyroptosis-related gene. **(C)** Principal component analysis of the transcriptome profiles of four pyroptosis-related gene signatures in the GSE87304 cohort. **(D)** Boxplots depicting the differences in

pyroptosis enzyme expression between the four pyroptosis gene signatures in the GSE87304 cohort. The asterisks represent the statistical *P* value (* $p<0.05$; ** $p<0.01$; *** $p<0.001$; **** $p<0.0001$). **(E)** Boxplots depicting the four pyroptosis gene signatures were distinguished by different signatures (immune-relevant signature, mismatch-relevant signature and stromal-relevant signature as indicated) in the GSE87304 cohort. The asterisks represent the statistical *P* value (* $p<0.05$; ** $p<0.01$; *** $p<0.001$; **** $p<0.0001$).

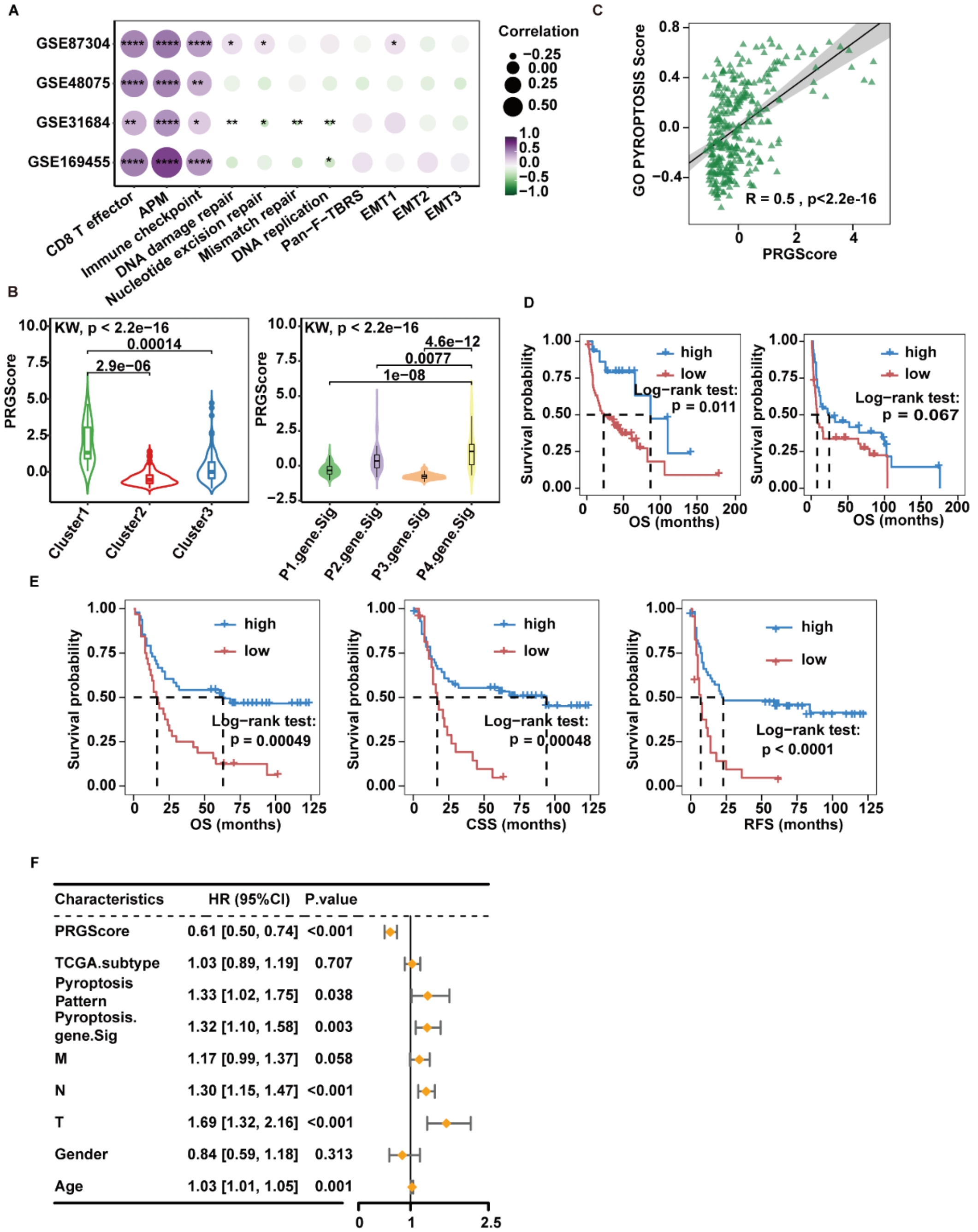

Supplementary Figure 5 | (A) The correlation between gene signature scores linked to EMT, immune checkpoint, mismatch repair, and immune activation and PRGScore analyzed in the GSE87304, GSE48075, GSE31684 and GSE169455 cohorts. The sizes of circles represent relevant Pearson correlation coefficients. The asterisks represent the statistical *P* value (* p<0.05; ** p<0.01; *** p<0.001; **** p<0.0001). **(B)** Differences in PRGScore among three pyroptosis patterns (left) and among four pyroptosis gene signatures (right) in GSE87304. The Kruskal-Wallis test was used to compare the significant differences between the three or four groups. **(C)** Scatter plots depicting the significantly positive correlation between PRGScore and GO pyroptosis signature score

in the GSE87304 cohort. **(D)** Survival analysis of the PRGScore-low and PRGScore-high groups in GSE48075 (left) and GSE31684 (right) using Kaplan-Meier curves. **(E)** Survival analysis for OS, CSS and RFS of the PRGScore-low and PRGScore-high groups in GSE169455 using Kaplan-Meier curves. **(F)** Multivariate Cox regression analysis for PRGScore in the TCGA-MIBC cohort summarized by forest plot.

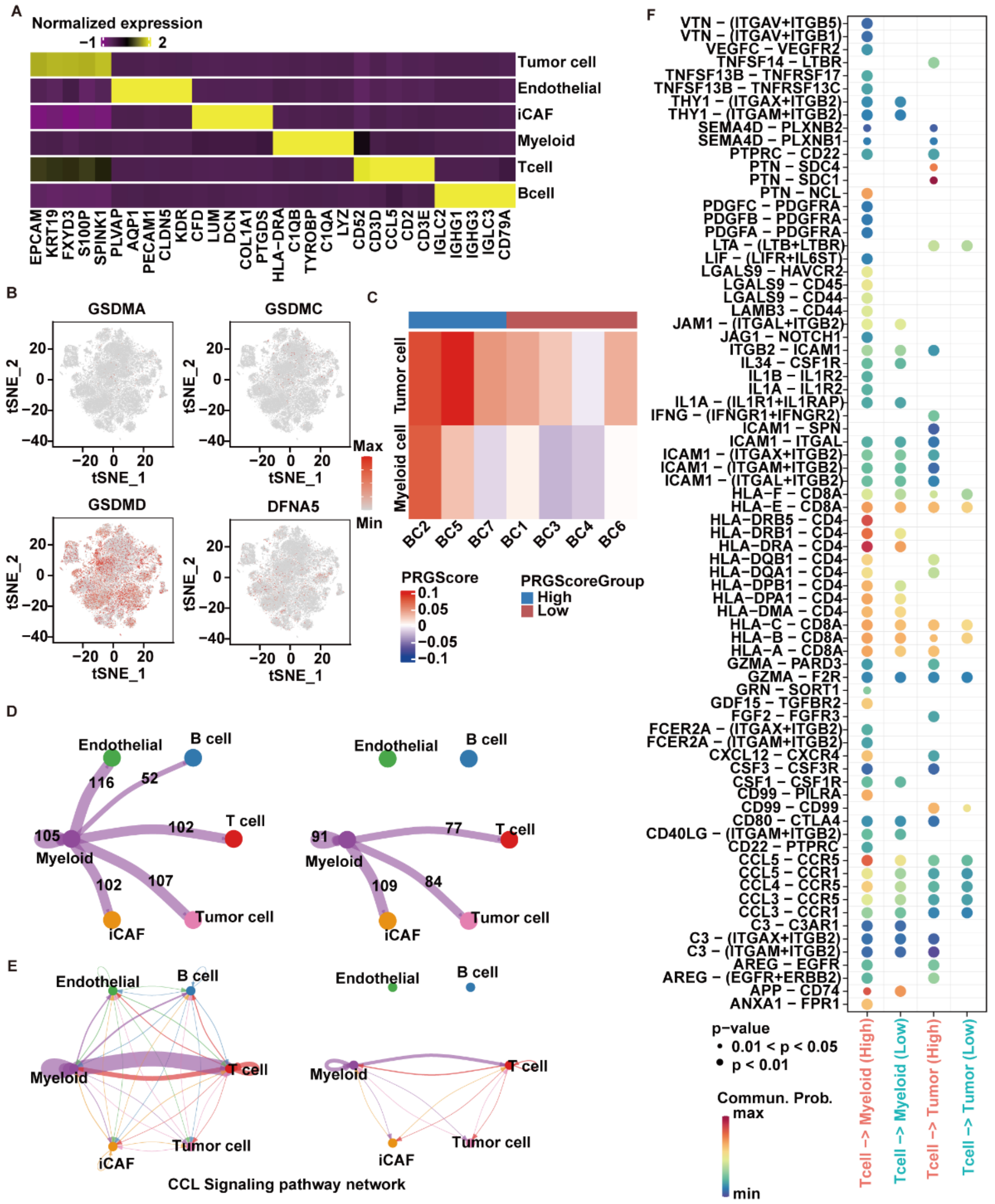

Supplementary Figure 6 | (A) Heatmap showing the expression of marker genes in the identified cell types. **(B)** t-SNE plots showing the expression levels of *GSDMA, GSDMC, GSDMD* and *DFNA5* for all the cell types **(C)** Heatmap showing the distribution in the PRGScore of tumor cells and myeloid cells (divided into 2 patterns) in seven bladder cancer samples. **(D)** Circos plots displaying putative ligand-receptor interactions between myeloid cells and other cell clusters from high-PRGScore (left) and low-PRGScore (right) group. The brand links pairs of interacting cell types, and corresponding number of events were labeled in the graph. **(E)** Circos plots showing the CCL signaling pathways between high-PRGScore (left) and low-PRGScore (right) group. **(F)** Comparison of the significant ligand-receptor pairs high-PRGScore and low-PRGScore group, which contribute to the signaling from T cells to Tumor cells and myeloid cells. Dot color reflects communication probabilities and dot size represents

computed p-values. Empty space means the communication probability is zero.

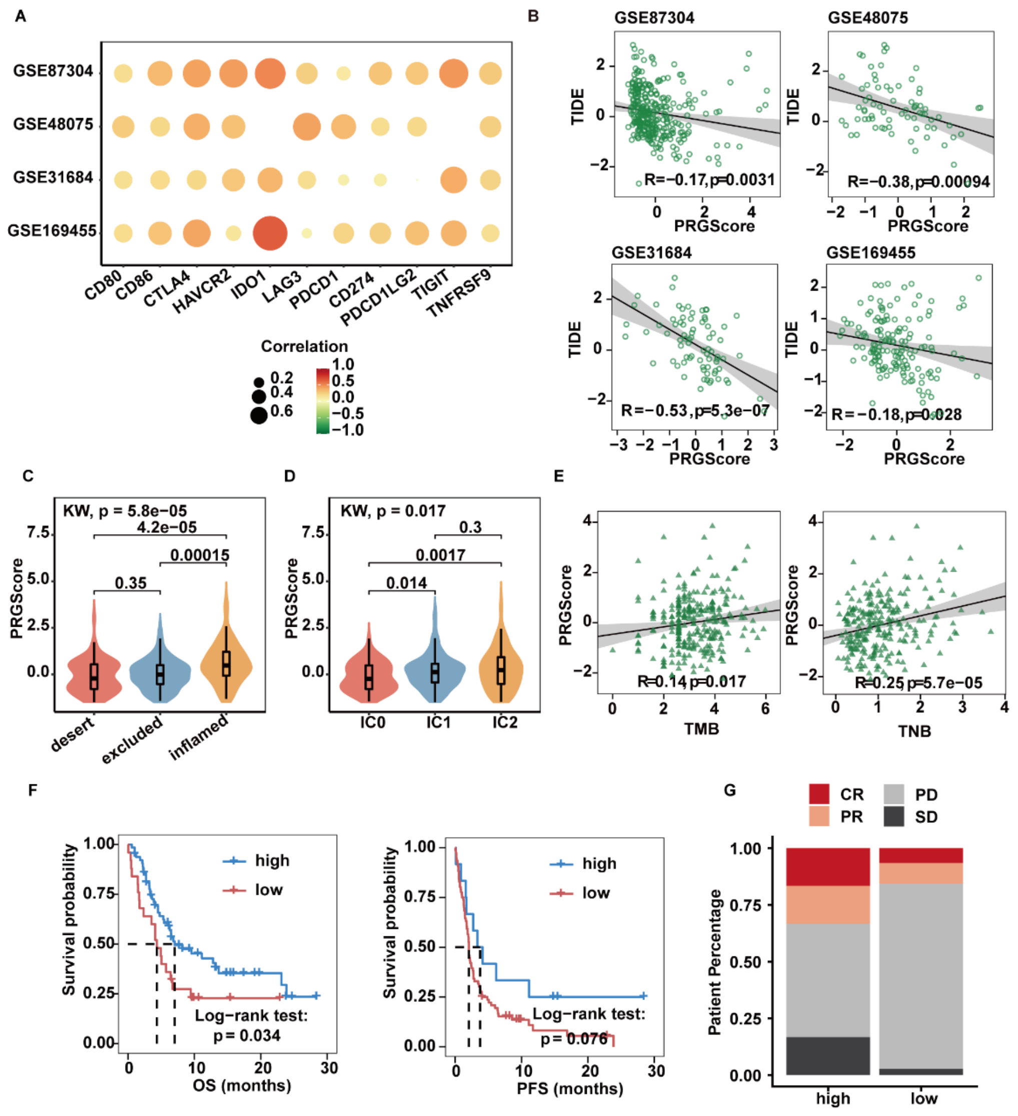

Supplementary Figure 7 | (A) Correlations between PRGScore and immune checkpoint gene expression in the GSE87304, GSE48075, GSE31684 and GSE169455 cohorts. **(B)** Scatter plots showing the significantly negative correlation between PRGScore and TIDE score in the GSE87304, GSE48075, GSE31684 and GSE169455 cohorts. The Pearson correlation between PRGScore and TIDE score is shown. **(C)** Differences in PRGScore among three immune subtypes in the IMvigor210 cohort. The Kruskal-Wallis test was used to compare the significant differences between the three groups. **(D)** PRGScores in immune cells (ICs) with different PD-L1 expression levels in the IMvigor210 cohort (IC0, < 1%; IC1, ≥ 1% and < 5%; IC2/3, ≥ 5%). The Kruskal-Wallis test was used to compare the significant differences between IC groups. **(E)**

Modest but significant correlations of PRGScore were shown with TMB (left) and TNB (right) in the IMvigor210 cohort. **(F)** Kaplan-Meier curves of OS (left) and PFS (right) for the high- and low-PRGScore groups in GSE176307. **(G)** The proportion of patients who responded to immune checkpoint blockade immunotherapy in the high or low PRGScore group in GSE176307.

Supplementary Table 1 | Basic information of MIBC datasets included in this study for identifying distinct pyroptosis regulation patterns and predicting prognosis value.

Supplementary Table 2 | Specific bladder cancer-related signature gene sets.

Supplementary Table 3 | Functional annotation of pyroptosis phenotype-related genes (Gene Ontology-Biological process).

Supplementary Table 4 | Functional annotation of pyroptosis phenotype-related genes (Kyoto Encyclopedia of Genes and Genomes).

Supplementary Table 5 | Prognostic analysis of 190 pyroptosis phenotype-related genes using a univariate Cox regression model.

Supplementary Table 6 | Immune-relevant signatures, mismatch-relevant signatures and stromal-relevant signature gene sets.

Supplementary Table 7 | BLCA-related drug target genes from the DrugBank database.
